# Open reduction and polyaxial plating for stemmed knee periprosthetic fractures: A case series

**DOI:** 10.1051/sicotj/2023022

**Published:** 2023-08-01

**Authors:** Diego Gonzalez-Morgado, Jose Vicente Andres-Peiro, Jordi Selga Marsa, Carlos Alberto Piedra Calle, Josep Francesc Nomdedeu Sancho, Jordi Teixidor Serra, Jordi Tomas Hernandez

**Affiliations:** 1 Orthopaedic Surgery Department, Hospital Universitari Vall d’Hebron, Universitat Autonoma de Barcelona Pg. Vall d'Hebron 119–129 08035 Barcelona Spain; 2 Orthopaedic Surgery Department, Orthopaedic Trauma Unit, Hospital Universitari Vall d’Hebron, Universitat Autonoma de Barcelona Pg. Vall d'Hebron 119–129 08035 Barcelona Spain

**Keywords:** Periprosthetic fracture, Femur, Locking plate, Inter-implant fracture, Revision arthroplasty

## Abstract

*Introduction*: Stemmed total knee arthroplasty (STKA) periprosthetic fractures (PPFs) are an emerging problem affecting frail patients. Their surgical fixation is challenging, due to intramedullary involvement and poor bone stock. Polyaxial locking plating has yielded good results in implant-related femur fractures. We hypothesized that this treatment would provide similar results for STKA PPFs. *Methods*: Retrospective analysis of consecutive patients with a femoral PPF or inter-implant fracture around a knee revision stem who had undergone open reduction and periprosthetic-specific polyaxial plate fixation. *Results*: We found 14 cases of mean age 85.4 years. Cerclages were used in 80% of cases. Fixation of a mean 8.6 cortices around the revision stem was achieved, with an overall screw density of 1:2 or 1:3. Four patients lost their ability to walk, while four experienced postoperative local complications. Bone healing was achieved in all except one who died during hospitalization. The 13 remaining survived the first year of follow-up. *Conclusion*: STKA PPFs are an emerging and challenging problem affecting frail patients. Treatment using polyaxial locking plates provides stable fixation allowing early mobilization despite high complication rates.

## Introduction

As the number of total knee arthroplasties (TKA) has increased, due to population aging and higher functional demand, the incidence of TKA complications and the number of revision TKA also have increased [1, 2]. Consequently, the number of stemmed TKA (STKA) periprosthetic fractures (PPFs) is rising [3].

Surgical management of distal femur PPFs with a stemmed implant is challenging in relation to: (1) bone healing being delayed in older patients who have impaired blood supply in their distal femur, due to previous surgeries and the presence of an intramedullary implant; (2) stable fixation being difficult to achieve due to poor bone stock and the existence of an often-cemented intramedullary implant; (3) prosthesis loosening that may precede the fracture; and (4) comorbidities in older patients, which can contribute to high postoperative complication rates and delay or even prevent patients from achieving their previous functional state.

The primary goal of surgical treatment in PPF is to restore limb length, axis, and rotation via stable fixation that allows early mobilization. Non-locking or monoaxial locking plates are associated with high failure rates when treating STKA PPFs [4, 5]. Previous works have shown that the consolidation rate for inter-implant, periprosthetic, and peri-implant femoral fractures ranges between 89% and 100% with periprosthetic polyaxial locking plates [5, 6]. To date, little information exists about the management of STKA PPFs [7, 8] and there are no publications evaluating the performance of polyaxial locking plating in this setting. Hence, lack of guidance and heterogeneity in treatment are the norm when dealing with this picture. We hypothesized that treating STKA PPFs with this implant could generate good results.

The primary aim of this paper was to report a series of implant-related fractures around a knee revision stem that were treated with a periprosthetic-specific polyaxial locking plate, as well as patients’ clinical outcomes, consolidation rate and complications. The secondary aim was to provide surgical guidance by describing the constructs used by our team for the fixation of these fractures.

## Material and methods

This retrospective case series study (level of evidence IV) was approved by our Institutional Review Board (PR(AT)28/2022) and writing was done under the PROCESS statement guidance. We included all patients with a femoral PPF or inter-implant fracture around a knee revision stem treated with a periprosthetic-specific polyaxial locking plate; specifically, the Non-Contact-Bridging (NCB) Periprosthetic Femur Plate System (Zimmer Biomet, USA). All patients were operated on consecutively at a single public university level-I trauma centre from May 2015 to May 2021 by a team of surgeons with considerable expertise managing patients with PPFs and followed-up for one year post-operatively. Exclusion criteria were pathological fracture, proximal femur fracture, and periprosthetic infection.

**Figure 1:**
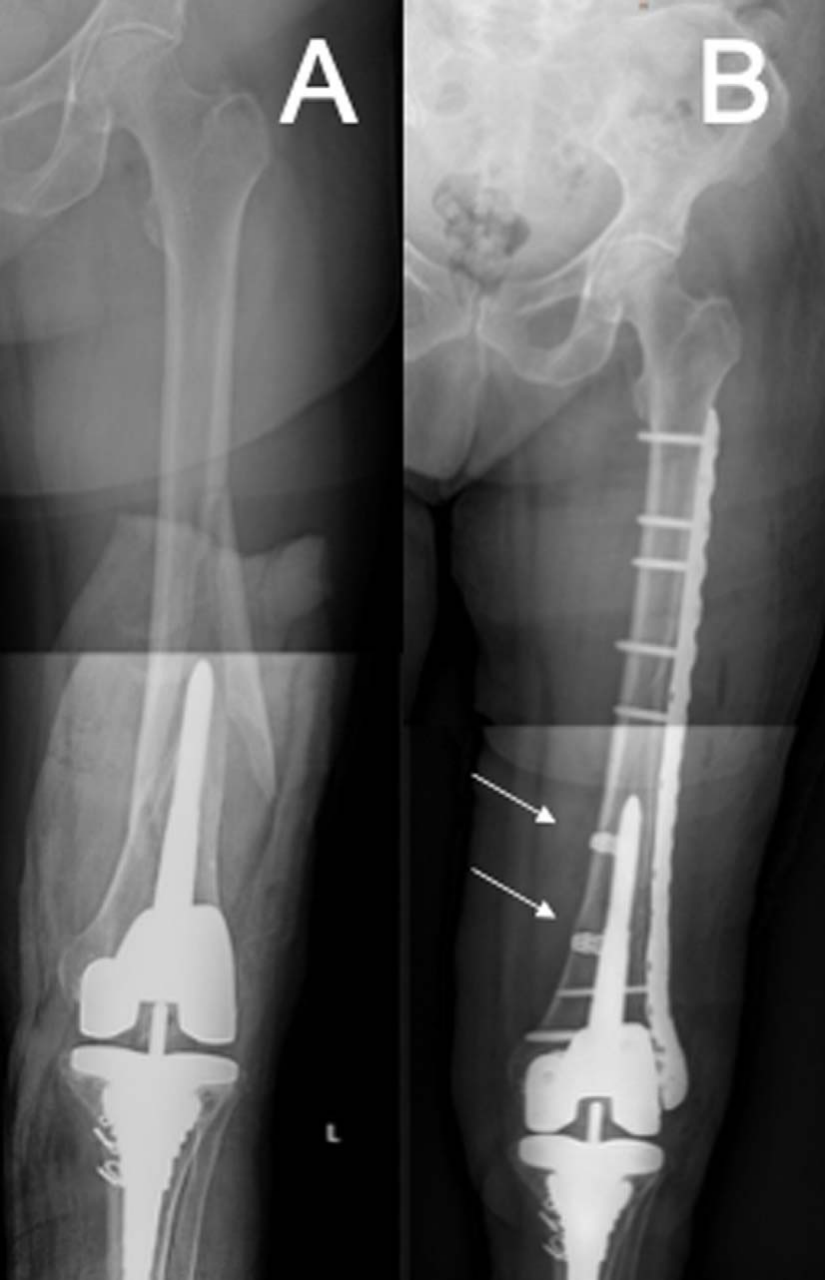
A) Long spiral periprosthetic fracture around a stemmed TKA. B) Cerclage cables were used as a reduction tool for long spiral or oblique fractures. SuperCable^®^ (Kinamed, USA) are shown.

Patients were admitted through our Emergency Department. As soon as safely possible for patients, surgical procedures were planned and performed. Surgery was performed with the patient in a supine position on a radiolucent table with the injured leg draped freely. The fracture was exposed using a lateral subvastus approach. Fracture reduction was achieved using manual traction and bone forceps. Cerclages replaced forceps for long oblique or spiral fracture patterns (Figure 1). The cerclages used were: Dall-Miles^®^ Cable System (Stryker, USA) and SuperCable^®^ (Kinamed, USA), a radiolucent polymer cable with a radiopaque titanium locking mechanism close-up. Then, a plate spanning the whole femoral diaphysis was inserted sub-muscularly and temporarily fixed with K-wires. Fracture reduction and implant positioning were continuously checked with the C-arm. We aimed for favourable stress modulation by: a working of length of two to three times the width of the femur at the level of the fracture and not less than the fracture extent; between six and eight cortices fixation around the stem, and 1:2-1:3 screw density [9].

Physical therapy – focussing on range of motion and strengthening – was started in the immediate postoperative period as well as aided walking as tolerated. Patients were discharged after they reached adequate clinical stability. Follow-up appointments were scheduled for six weeks, and for three, six and 12 months after surgery. Thorough clinical and radiological examinations were completed at all follow-up appointments.

All relevant data on patients’ baseline characteristics, injuries, treatment, and follow-up assessments were entered into a codified Microsoft Excel database from the institutional database for medical records. The evaluation of each radiological image was performed once by one designated investigator (DGM) using the RAIM Viewer software (Corporació Sanitària Parc Tauli, Spain). Fractures were classified using the Unified Classification System for Periprosthetic Fractures (UCPF) [10]. Operating time, transfusion requirements, use of cerclages, number of cortices fixed around the stem, overall screw density, and working length were recorded as treatment variables. Screw density was calculated by dividing the number of screws by the number of plate holes. Working length was defined as the distance between the two innermost screws on either side of the fracture [11]. Final ambulatory status, one-year surveillance, and complications related to surgical site infection, non-union or fixation failure were recorded during follow-up visits. Bone healing was defined as bridging of the fracture site and the absence of pain at the fracture site during weight bearing. The time threshold chosen to diagnose non-union was 12 months [12].

Since no control group was available and the sample size limited, only descriptive statistics were calculated, with categorical variables summarized as counts and percentages, and continuous variables as means and standard deviations or medians and ranges, as appropriate. Stata 14.2 software (StataCorp, USA) was used for such analysis.

## Results

About 191 implant-related femur fractures were operated on during the recruitment period. 17 (8.9%) of them were STKA PPFs and 14 met the inclusion criteria. We excluded two patients treated with a distal femoral replacement and one who died prior to surgery. There were 2 men and 12 women, of mean age 85.4 ± 8.1 years. All patients had significant comorbidities according to the American Society of Anesthesiologists physical status classification (ASA ≥ II), being severe (ASA ≥ III) in eight. Mean body mass index was 31.8 ± 6.9. Pre-operatively, two patients were non-ambulatory and six ambulated with assistive devices.

All injuries were caused by falls from a standing height or transfers. We found nine PPFs and five inter-implant fractures, all of them at the stem’s tip or around it. According to the UCPF classification, there were 4 V3B1 fractures (stable prosthesis and good bone stock), 2 V3B2 fractures (loose prosthesis and good bone stock), 3 V3B3 (loose prosthesis and poor bone stock) and 5 V3D (dividing the bone between two implants). The proximal implants were 2 metaphyseal stems for primary hip replacement, 2 cephalomedullary nails, and 1 dynamic hip screw.

The median time from revision replacement to PPF was 7.9 years (range 1–18 years). The indication for revision was mechanical loosening in 6 cases, painful or unstable arthroplasty in 5, periprosthetic knee fracture in 2 and periprosthetic joint infection in 1. The femoral stem was cemented in 8 cases (57%).

**Figure 2:**
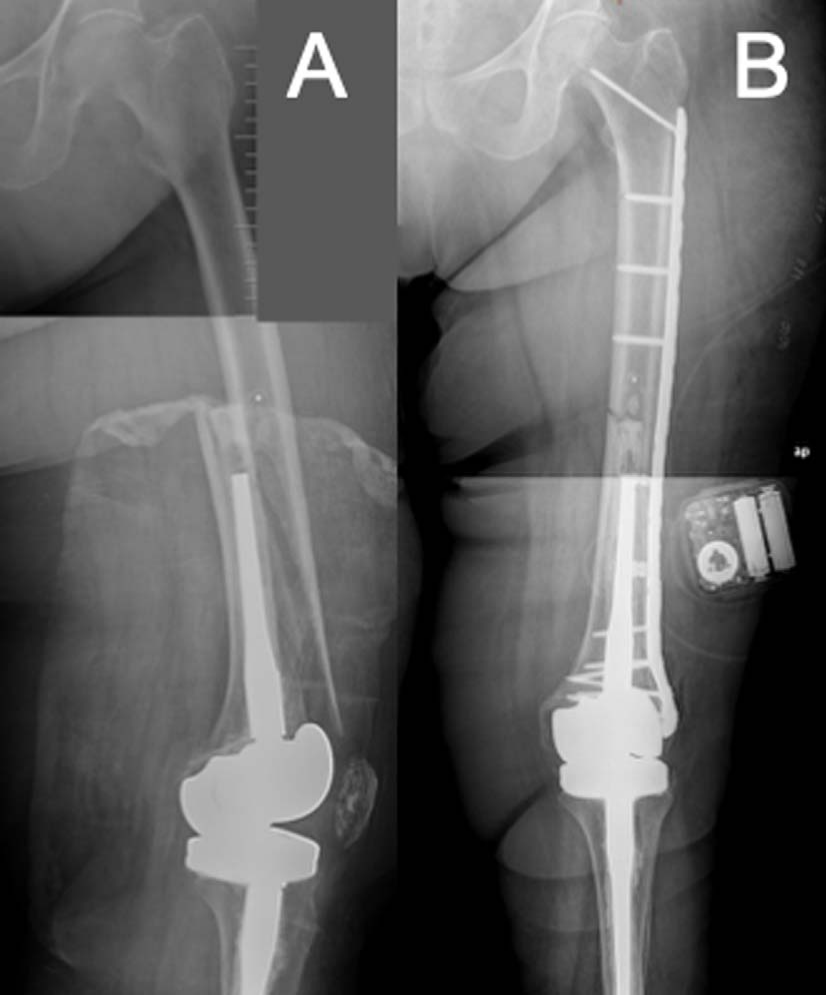
A) Long spiral periprosthetic fracture around a stemmed TKA. B) Flexible fixation with well-spaced screws and an overall screw density between 1:2 and 1:3.

Cerclages were used as an additional method of reduction and stabilization in 11 (80%) cases. Mean fixation of 8.6 ± 2.2 cortices around the revision stem was achieved with 3.6 ± 1.5 bicortical and 1.4 ± 1.5 monocortical screws, with an overall screw density of 1:2 for seven and 1:3 for seven cases (50% each) (Figure 2B). Mean working length was 123.4 mm (range 0–257.0 mm). Mean operating time was 129.7 ± 22.2 min and the median transfusion requirement was two units of packed red blood cells (range 1–10).

The mean length of hospitalization was 22.8 ± 15.9 days and the mean time from admission to surgery 5.9 ± 4.2 days. One death occurred during hospitalization, related to congestive heart failure. 10 of the 13 survivors (76.9%) were referred to a nursing care facility at discharge. Six patients (42.9%) were unable to walk, an increase of 4 from pre-operatively. Among that 8 who were ambulatory post-operatively, 3 (37.5%) used a walker. Full-weight-bearing began at an average of 5.6 ± 3.9 weeks after the fixation procedure.

Four patients (28.6%) developed local complications postoperatively. Three of these patients had an infection that healed with debridement and proper antibiotics. One patient experienced failure of fixation requiring distal femoral replacement. Bone healing was achieved in all patients who survived at a median time of 32 weeks (range 11–50). All patients, except the one who died during hospitalization, survived the first year of follow-up (7% mortality).

## Discussion

STKA PPFs are an emerging problem, so information about their management is still scarce. Periprosthetic polyaxial locking plates seem to be a promising option, as previous studies have shown for similar fractures [5, 6]. In our population of 191 femur implant-related fractures, 7.3% experienced a fracture around a STKA that was managed by lateral polyaxial locking plating. They were mainly obese, frail and functionally-impaired; so the surgical treatment yielded a high rate of postoperative complications.

This study has many limitations: retrospective evaluation of medical records, radiological assessment by only one observer preventing interrater agreement evaluation, short sample size and the fracture pattern heterogeneity. Despite this, the lack of guidance on the management of this particular picture and the treatment of all injuries applying a standardized protocol and the thorough description of the surgical technique and results; put in value the messages of this paper.

STKA PPFs are characterized by the presence of an intramedullary implant in a fragile bone, so the achievement of a stable fixation that allows early and painless mobilization may be challenging [4, 5]. Different surgical treatments have been described for their management. Treatment with distal femoral replacement tends to allow earlier weight bearing relative to plate fixation, but has been linked to as high as a 64% rate of postoperative complications [13], revision rates as high as 27.5% at 10 years [14], and mortality rates up to 36% at one year [15]. In terms of peri-implant fracture fixation, non-locking plates have resulted in complication rates up to 53%, while locking plates offer stable fixation of osteoporotic fractures, allowing immediate weight-bearing, as tolerated, with low implant failure and non-union rates [16]. However, the presence of a stem makes it difficult to achieve stable fixation [6]. Hoon Shin presented a series of STKA PPFs in which six out of 15 patients were treated by dual-locking plates with good results [8]. Dual plating is generally recommended for low metaphyseal distal femoral fracture, especially with medial comminution, and implant-related complications associated to this fixation technique cannot be ignored [17]. Moreover, STKA PPFs are often found at the tip of the stem or around it, as our results showed, and dual plating may not be necessary. The problem of achieving a stable fixation around the stem can also be addressed with periprosthetic-specific polyaxial locking plates that permit bi-cortical screw fixation around the prosthesis with a single implant [5, 6, 18]. Distinctly, the plate system used in our series has off-set holes that allow screw placement around the stem providing stable bicortical fixation. Consolidation rates for inter-implant, periprosthetic and peri-implant femoral fractures range between 89% and 100% with this plate system [5, 6, 18], similar to what we observed in our series. Thus, we strongly recommend single-plating with these implants for fragility PPFs with a well-fixed stem-prosthesis, considering distal femoral replacement for grossly loose implant in low-physical-demand patient.

**Figure 3:**
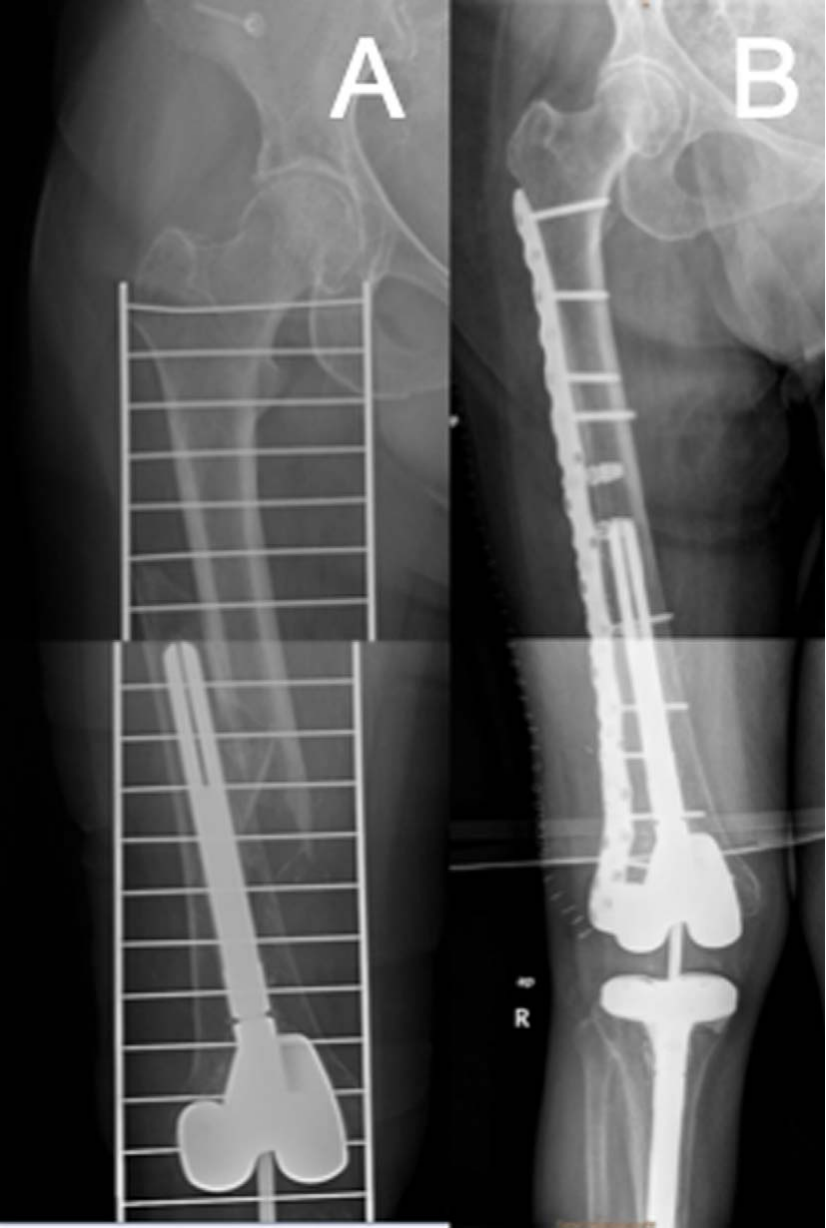
A) Oblique periprosthetic fracture around a stemmed TKA. B) Fixation with a long plate from the knee metaphysis to lesser trochanter with large fracture spanning. Fixation around the stem with at least six cortices.

Surgical techniques were performed aimed at achieving favourable stress modulation via a flexible fixation technique without sacrificing strength. This can be obtained using longer plates, with well-spaced screws, that span the whole femoral diaphysis, thereby increasing flexibility while avoiding stress risers that may jeopardize peri-implant fractures (Figure 3B); and a hybrid fixation technique with locking screws in the metaphyseal area and non-locking screws in the diaphysis [11]. Compared to the eight-cortex fixation approach around the stem that Ruchholtz et al. described [6], we aimed for at least six cortices (Figure 3C), considering the results reported previously by Peiró et al. with this implant while treating unstable proximal femur fractures above a knee revision stem [19]. In most of our patients, cerclage wiring was used as a reduction tool and not fixed to the plate. This is because, although its use close to a fracture has traditionally been discouraged, its application helps with fracture reduction and fixation when it is placed without significant periosteal blood disruption [20, 21].

**Figure 4:**
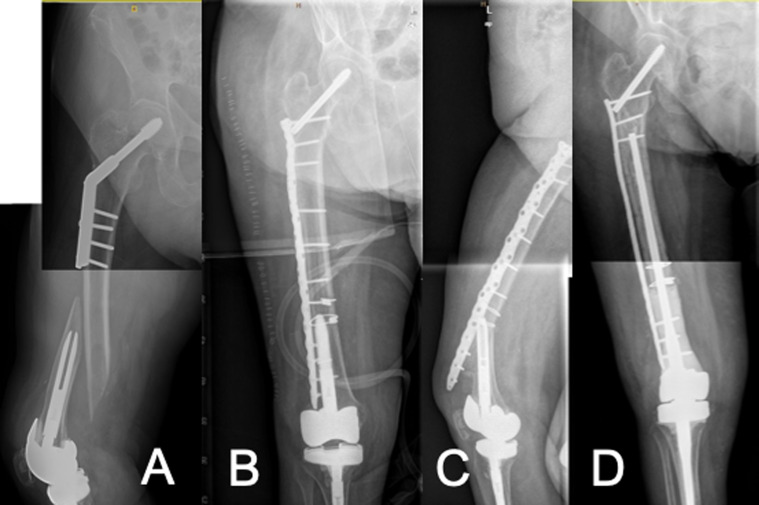
A) Oblique inter-implant fracture around a stemmed TKA with proximal dynamic hip screw device. B) Improper fixation with insufficient cortex fixation around the stem. C) Fixation failure. D) Distal femoral replacement for fixation failure treatment.

Up to a 48% rate of complications and reoperations treating periprosthetic femur fractures have been reported in the literature [22], akin to what we observed in our series. Non-union rates have ranged from 0% to 33% utilizing monoaxial locking plate designs in some series [22–24]. Lower rates have been found with polyaxial locking plates [5, 6], ranging between 5% and 11%. In our series, we experienced no non-union, but one patient had a plate pull out due to improper fixation: just four-cortex fixation with four monocortical screws was achieved around the STKA implant (Figure 4). Mortality rates after distal femoral fractures (native or periprosthetic) in patients above the age of 60 have been shown to be 25% at one year, similar to the rate for hip fractures [27, 28]. We had a lower mortality rate (just one death in 14 patients), probably due to our small sample size. The main reasons for our long length of hospitalization were related to operating room availability, social care issues and the slow recovery from surgery of our fragile patients. Long length of stay is associated with increased morbidity, mortality, and cost in the treatment of PPFs. In contrast to our level-1 trauma centre organization, healthcare infrastructures with a central organization and secondary units function well when managing of PPFs decreasing length of hospital stay [28]. This is relevant considering the increasing burden of PPFs.

Final ambulatory status after a distal femur PPF with a STKA is typically quite impaired, as our results suggest. One explanation for this is that these patients are, by nature, very fragile, have a history of functional impairment, and have usually had more than one previous surgery in the distal femur. This may explain why although our patients were allowed to weight-bearing as tolerated after surgery, it took over a month to full-weight-bearing for many of them. Similar results treating peri-implant femur fractures with this specific implant have been described for another series, in which 16% of the patients were non-ambulatory after their fracture and 47.8% had severe walking limitations [6].

## Conclusions

Stemmed TKA periprosthetic fractures are an emerging problem that generally affects already-frail patients. Their surgical fixation poses a challenge due to their intramedullary involvement and patients’ poor bone stock. Treating these fractures using polyaxial locking periprosthetic-specific plates appears to be a promising option which provides stable fixation and, by doing so, early mobilization. Nonetheless, high complication rates are to be expected. Future larger and prospective studies are needed to properly standardize the management of this specific fracture pattern.

## Ethics approval

We declare that this study was approved by our Institutional Review Board under the code PR(AT)28/2022.

## Consent to participate

Not applicable. This study was approved by our Institutional Review Board under the code PR(AT)28/2022.

## Consent to publish

Not applicable. This study was approved by our Institutional Review Board under the code PR(AT)28/2022.

## Author contributions

All authors contributed to the study conception and design. Material preparation, data collection was performed by JTS, JSM, JFNS and CAPC. Analysis was performed by JVAP. The first draft of the manuscript was written by DGM. Supervision and guidance were performed by JTH. All authors commented on previous versions of the manuscript. All authors read and approved the final manuscript.

## Availability of data and materials

Sample date will be available under request.

## References

[1] Kurtz S, Ong K, Lau E (2007) Projections of primary and revision hip and knee arthroplasty in the United States from 2005 to 2030. J Bone Joint Surg Am 89, 780–785. 17403800 10.2106/JBJS.F.00222

[2] Parvizi J, Jain N, Schmidt AH (2008) Periprosthetic knee fractures. J Orthop Trauma 22, 663–671. 18827599 10.1097/BOT.0b013e31816ed989

[3] Meek RMD, Norwood T, Smith R (2011) The risk of peri-prosthetic fracture after primary and revision total hip and knee replacement. J Bone Joint Surg Br 93, 96–101. 21196551 10.1302/0301-620X.93B1.25087

[4] Solarino G, Vicenti G, Moretti L (2014) Interprosthetic femoral fractures – A challenge of treatment. A systematic review of the literature. Injury 45, 362–368. 24119494 10.1016/j.injury.2013.09.028

[5] Hoffmann MF, Lotzien S, Schildhauer TA (2016) Clinical outcome of interprosthetic femoral fractures treated with polyaxial locking plates. Injury 47, 934–938. 26792022 10.1016/j.injury.2015.12.026

[6] Ruchholtz S, El-Zayat B, Kreslo D (2013) Less invasive polyaxial locking plate fixation in periprosthetic and peri-implant fractures of the femur – a prospective study of 41 patients. Injury 44, 239–248. 23219240 10.1016/j.injury.2012.10.035

[7] Ebraheim NA, Kelley LH, Liu X (2015) Periprosthetic distal femur fracture after total knee arthroplasty: a systematic review. Orthop Surg 7, 297–305. 26790831 10.1111/os.12199 PMC6583744

[8] Shin JH, Chang MJ, Kang S-B (2019) Management and clinical outcomes of periprosthetic fractures after total knee arthroplasty with a stem extension. Medicine (Baltimore) 98, e16088. 31232950 10.1097/MD.0000000000016088 PMC6636976

[9] Patsiogiannis N, Kanakaris NK, Giannoudis PV (2021) Periprosthetic hip fractures: an update into their management and clinical outcomes. EFORT Open Rev 6, 75–92. 33532088 10.1302/2058-5241.6.200050 PMC7845569

[10] Unified Classification System for Periprosthetic Fractures (UCPF). (2018) J Orthop Trauma 32 Suppl 1:S141–S144. 29256962 10.1097/BOT.0000000000001068

[11] Beltran MJ, Collinge CA, Gardner MJ (2016) Stress modulation of fracture fixation implants. J Am Acad Orthop Surg 24, 711–719. 27579811 10.5435/JAAOS-D-15-00175

[12] Cunningham BP, Brazina S, Morshed S, Miclau T (2017) Fracture healing: A review of clinical, imaging and laboratory diagnostic options. Injury 48(Suppl 1), S69–S75. 28483359 10.1016/j.injury.2017.04.020

[13] Mortazavi SMJ, Kurd MF, Bender B (2010) Distal femoral arthroplasty for the treatment of periprosthetic fractures after total knee arthroplasty. J Arthroplasty 25, 775–780. 20171053 10.1016/j.arth.2009.05.024

[14] Wyles CC, Tibbo ME, Yuan BJ (2020) Long-term results of total knee arthroplasty with contemporary distal femoral replacement. J Bone Joint Surg 102, 45–51. 31596808 10.2106/JBJS.19.00489

[15] Windhager R, Schreiner M, Staats K, Apprich S (2016) Megaprostheses in the treatment of periprosthetic fractures of the knee joint: indication, technique, results and review of literature. Inter Orthop (SICOT) 40, 935–943. 10.1007/s00264-015-2991-4 26404093

[16] Smith WR, Stoneback JW, Morgan SJ, Stahel PF (2016) Is immediate weight bearing safe for periprosthetic distal femur fractures treated by locked plating? A feasibility study in 52 consecutive patients Patient Saf Surg 10, 26. 27980675 10.1186/s13037-016-0114-9 PMC5142343

[17] Steinberg EL, Elis J, Steinberg Y (2017) A double-plating approach to distal femur fracture: a clinical study. Injury 48, 2260–2265. 28768571 10.1016/j.injury.2017.07.025

[18] Erhardt JB, Grob K, Roderer G (2008) Treatment of periprosthetic femur fractures with the non-contact bridging plate: a new angular stable implant. Arch Orthop Trauma Surg 128, 409–416. 17639435 10.1007/s00402-007-0396-6

[19] Peiró JVA, Ruiz MJ, Hernández JT (2021) The inverted Vancouver C fracture. Case series of unstable proximal femur fractures above a knee revision stem treated by short cephalomedullary nail and lateral submuscular overlapping plate. Eur J Orthop Surg Traumatol 31, 193–198. 32691167 10.1007/s00590-020-02738-8

[20] Perren SM, Fernandez Dell’Oca A, Lenz M, Windolf M (2011) Cerclage, evolution and potential of a Cinderella technology. An overview with reference to periprosthetic fractures. Acta Chir Orthop Traumatol Cech 78, 190–199. 21729634

[21] Apivatthakakul T, Phaliphot J, Leuvitoonvechkit S (2013) Percutaneous cerclage wiring, does it disrupt femoral blood supply? A cadaveric injection study Injury 44, 168–174. 23164676 10.1016/j.injury.2012.10.016

[22] Zuurmond RG, van Wijhe W, van Raay JJAM, Bulstra SK (2010) High incidence of complications and poor clinical outcome in the operative treatment of periprosthetic femoral fractures: An analysis of 71 cases. Injury 41, 629–633. 20236641 10.1016/j.injury.2010.01.102

[23] Platzer P, Schuster R, Luxl M (2011) Management and outcome of interprosthetic femoral fractures. Injury 42, 1219–1225. 21176899 10.1016/j.injury.2010.08.020

[24] Soenen M, Migaud H, Bonnomet F (2011) Interprosthetic femoral fractures: analysis of 14 cases. Proposal for an additional grade in the Vancouver and SoFCOT classifications. Orthop Traumatol Surg Res 97, 693–698. 21982823 10.1016/j.otsr.2011.07.009

[25] Hou Z, Moore B, Bowen TR (2011) Treatment of interprosthetic fractures of the femur. J Trauma 71, 1715–1719. 22182879 10.1097/TA.0b013e31821dd9f1

[26] Michla Y, Spalding L, Holland JP, Deehan DJ (2010) The complex problem of the interprosthetic femoral fracture in the elderly patient. Acta Orthop Belg 76, 636–643. 21138219

[27] Streubel PN (2013) Mortality after periprosthetic femur fractures. J Knee Surg 26, 27–30. 23393056 10.1055/s-0033-1333905

[28] Mudiganty S, Hughes L, Choudry Q, Bokhari A (2022) Managing periprosthetic fractures – a review of the hub and spoke model. SICOT J 8, 2. 35040775 10.1051/sicotj/2022001 PMC8765126

